# From Cognitive Bias Toward Advanced Computational Intelligence for Smart Infrastructure Monitoring

**DOI:** 10.3389/fpsyg.2022.846610

**Published:** 2022-03-24

**Authors:** Meisam Gordan, Ong Zhi Chao, Saeed-Reza Sabbagh-Yazdi, Lai Khin Wee, Khaled Ghaedi, Zubaidah Ismail

**Affiliations:** ^1^Department of Civil Engineering, Universiti Malaya, Kuala Lumpur, Malaysia; ^2^Department of Civil Engineering, K. N. TOOSI University of Technology, Tehran, Iran; ^3^Department of Mechanical Engineering, Universiti Malaya, Kuala Lumpur, Malaysia; ^4^Department of Biomedical Engineering, Universiti Malaya, Kuala Lumpur, Malaysia

**Keywords:** cognitive bias, infrastructure health monitoring, bridge monitoring, artificial intelligence, safety

## Abstract

Visual inspections have been typically used in condition assessment of infrastructure. However, they are based on human judgment and their interpretation of data can differ from acquired results. In psychology, this difference is called cognitive bias which directly affects Structural Health Monitoring (SHM)-based decision making. Besides, the confusion between condition state and safety of a bridge is another example of cognitive bias in bridge monitoring. Therefore, integrated computer-based approaches as powerful tools can be significantly applied in SHM systems. This paper explores the relationship between the use of advanced computational intelligence and the development of SHM solutions through conducting an infrastructure monitoring methodology. Artificial Intelligence (AI)-based algorithms, i.e., Artificial Neural Network (ANN), hybrid ANN-based Imperial Competitive Algorithm, and hybrid ANN-based Genetic Algorithm, are developed for damage assessment using a lab-scale composite bridge deck structure. Based on the comparison of the results, the employed evolutionary algorithms could improve the prediction error of the pre-developed network by enhancing the learning procedure of the ANN.

## Introduction

Many SHM approaches based on visual inspections have been carried out to assess the local or global condition of infrastructure (Gordan et al., 2020b). However, they have many limitations. For example, they are laborious and based on human judgment (Dong and Catbas, 2021). In other words, the interpretation of data using visual inspections can differ from outcomes acquired from a rational process (Korteling et al., 2018). In psychology, this difference is called cognitive bias which directly affects SHM-based decision making (Berthet, 2021; Verzobio et al., 2021). Besides, emotional or neutral interpretations cause decision biases (Schoth and Liossi, 2017). In bridge monitoring, the confusion between condition state and safety of a bridge is another example of cognitive bias which has been reported by Zonta et al. (2007).

Computer-based automation of sensing, analysis, and decision making in different applications is becoming important for learning, pattern recognition, and computation by using artificial intelligence techniques (Sheridan, 2019). In the same direction, to overcome the aforesaid decision errors or biases, advanced computational intelligence techniques are gaining increasing attention for developing the SHM strategies. For instance, data mining techniques (Gordan et al., 2017, 2018, 2021c; Tan et al., 2021), cloud computing (Abdulkarem et al., 2020), and deep learning (Wang et al., 2021) have recently been used in SHM. Both AI and machine learning are also considered as emerging technologies in the 2020s. In recent years, ANNs have obtained extensive attention in SHM because of their extreme pattern recognition capacity (Gordan et al., 2021a). Besides, these days several metaheuristic-based biological algorithms exist, e.g., GA (Daves et al., 2021), ant colony optimization (Soheili et al., 2021), grey wolf optimization (Noshadi et al., 2015), particle swarm optimization (Xue et al., 2013), artificial immune algorithm (Poteralski et al., 2013), artificial bee colony algorithm (Li et al., 2021), and firefly algorithm (Zhou et al., 2014). The GA is one of the most popular representatives of evolutionary algorithms for solving global optimization problems (Miller and Ziemiański, 2020). This AI algorithm which was inspired by Darwin’s theory is capable of improving the generalization performance of artificial models (Elbaz et al., 2021). As known, metaheuristic evolutionary algorithms are not limited to biological evolution. For example, an evolutionary strategy, named as Imperialist Competitive Algorithm (ICA), has been introduced recently which is based on humans’ social political evolution (Atashpaz-Gargari and Lucas, 2007). This metaheuristic algorithm has also shown its extreme global optimal solution for optimization problems by providing fast convergence speed as well as great performance (Gordan et al., 2020b).

Based on the above explanations, this study aims to explore the relationship between the use of advanced computational intelligence and the development of SHM schemes through conducting a smart infrastructure monitoring methodology. Therefore, the methodology of the paper is presented in Section 2. The performed case study is presented in this section. Here, the insight details of developed AI algorithms are also explained in Section 2. The results of the developed networks are discussed in Section 3. The performance of the patterns is also compared in this section. Future work direction is addressed in Section 4. Then, the important conclusions are drawn in Section 5.

## Methodology

The methodology of this study is divided into two categories, comprising operating techniques and diagnosis techniques. Operating techniques are referring to those methods that are used to generate the datasets, i.e., inverse analysis. Diagnosis techniques are referring to the methods and procedures that are employed to analyze the datasets, such as the advanced computational intelligence techniques, i.e., artificial neural network (ANN), hybrid ANN-based Imperial Competitive Algorithm (ANN-ICA), and hybrid ANN-based Genetic Algorithm (ANN-GA).

### Case Study

**Figure 1:**
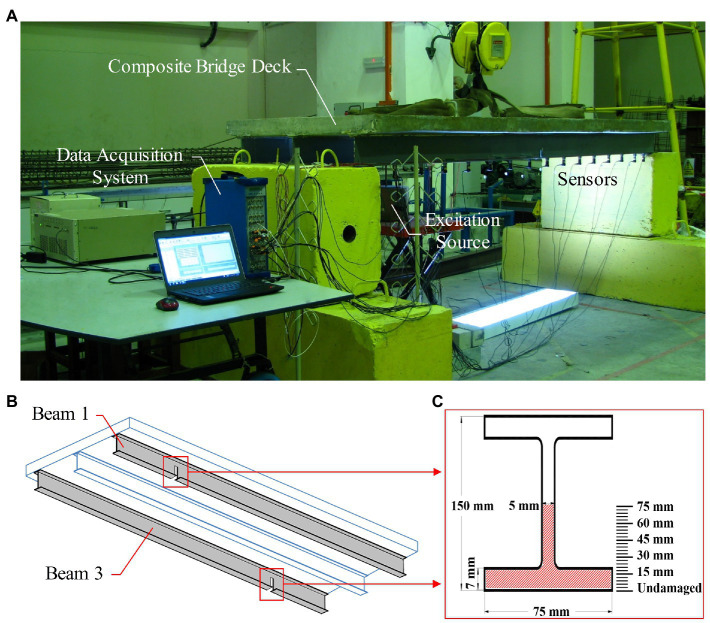
**(A)** Experimental test of the lab-scale bridge deck, **(B)** Damage locations, and **(C)** Damage severities.

The common span length and girder spacing of a typical steel multi-girder composite bridge are 25 m to 30 m and 3.5 m to 4 m, respectively. A scaled model of a typical multi-girder composite bridge deck with a 1:10 scale ratio was fabricated in the heavy structure laboratory of the Department of Civil Engineering, University of Malaya. The model consists of three Universal steel beams attached to a concrete slab using shear stud connectors. The length of the specimen is 3,200 mm including 100 mm at both support ends. The overall dimensions of steel beams comprise the flange width of 75 mm, section depth of 150 mm, and thickness of 7 mm and 5 mm for the flange and web, respectively. The Young’s Modulus of the steel is 2.1^*^1011 kg/m^2^, with Poisson’s ratio of 0.3 and density of 7,850 kg/m^3^. The dimension of the slab includes the width of 1,200 mm, the depth of 100 mm, and length of 3,200 mm. The materials used in this work are cement, fine aggregates, silica fume, water, and super-plasticizer. Poisson’s ratio, density, and strength of concrete are 0.2, 2,400 kg/m^3^ and 37.43 MPa, respectively. Reinforcement in concrete slab is welded wire mesh. Its diameter is 5 mm with 100 mm by 100 mm spacing. The concrete cover for mesh from below is 30 mm. Full composite action between the concrete slab and steel I-beams is modeled using 16 shear stud connectors which are installed on each I-beam. The diameter of each stud is 16 mm with 200 mm center to center spacing and height of 75 mm. The nuts are welded on top of beam flange and the bolts are firmly tightened to the nuts. Figure 1A demonstrates the vibration test setup of the scaled composite slab-on-girder model. Experimental modal analysis of the undamaged specimen was carried out as a benchmark. Subsequently, several damage scenarios were imposed on the test structure, as shown in Figures 1B,C and Table 1. To aid the aim, a total of 25 damage depths were conducted from 3 mm severity up to 75 mm depth. In detail, the increment of damage depth was 3 mm. The outputs of the aforesaid process are employed in the function of input for the soft computing procedure.

**Table 1:** The detailed explanation of structural damage scenarios.

Damage case	Description			
	Damage type	Damage location	Damage severity	Damage width
Healthy state	No damage (Reference)	–	–	–
Damaged state	Notch cutting	One-quarter span of beam 1 and three-quarter span of beam 3	3–75 mm	5 mm

### Advanced Computational Intelligence

ANN was introduced in 1980s, uses human brain simulation. This approach is a strong self-organizing computational algorithm inspired by the development of biological neurons models (Gordan et al., 2020a). A basic biological neuron comprises a cell body, dendrites, axons, and synapses. Input signals are entered to the cell body by the dendrites. Synapses are the point contacts between dendrites and axons. The output signals are transferred to other neurons by axons (Karacı and Arıcı, 2014). ANN is capable of solving difficult and nonlinear functions through self-organization, pattern recognition, and functional approximation (Gordan et al., 2020a). This technique has the ability to identify patterns between input and output variables (Tan et al., 2020). Hence, it has attracted considerable attention in engineering systems. For example, various studies have been done on the systems that combine ANN with other data mining methods such as Bayesian ANN (Lam and Beck, 2006; Jiang and Mahadevan, 2008) and fuzzy networks (Wen et al., 2007). However, ANNs have uncertainty in assigning weights to connections between layers. This shortcoming can reduce the accuracy of the network. In order to solve this problem, optimization-based methods can be employed to improve the training phase of the network. To this end, two hybrid techniques are developed in this study by combining the pre-developed network with Imperial Competitive Algorithm (ICA) and Genetic Algorithm (GA). ICA is one of the most recent evolutionary algorithms which was introduced by Atashpaz-Gargari and Lucas (2007). This population-based approach is based on humans’ socio-political evolution and it has been successfully applied in several optimization problems (Ebrahimi et al., 2014). Therefore, this computing algorithm showed its superior ability to acquire the global optima (Hajihassani et al., 2015). GA, another metaheuristic algorithm, was introduced in 1970s which has been also used in many optimization problems. Both evolutionary algorithms work with random populations to find the solution. In ICA, the imperialist with colonized countries form an empire. Likewise, in GA, chromosomes include a group of genes.

## Results and Discussion

**Figure 2:**
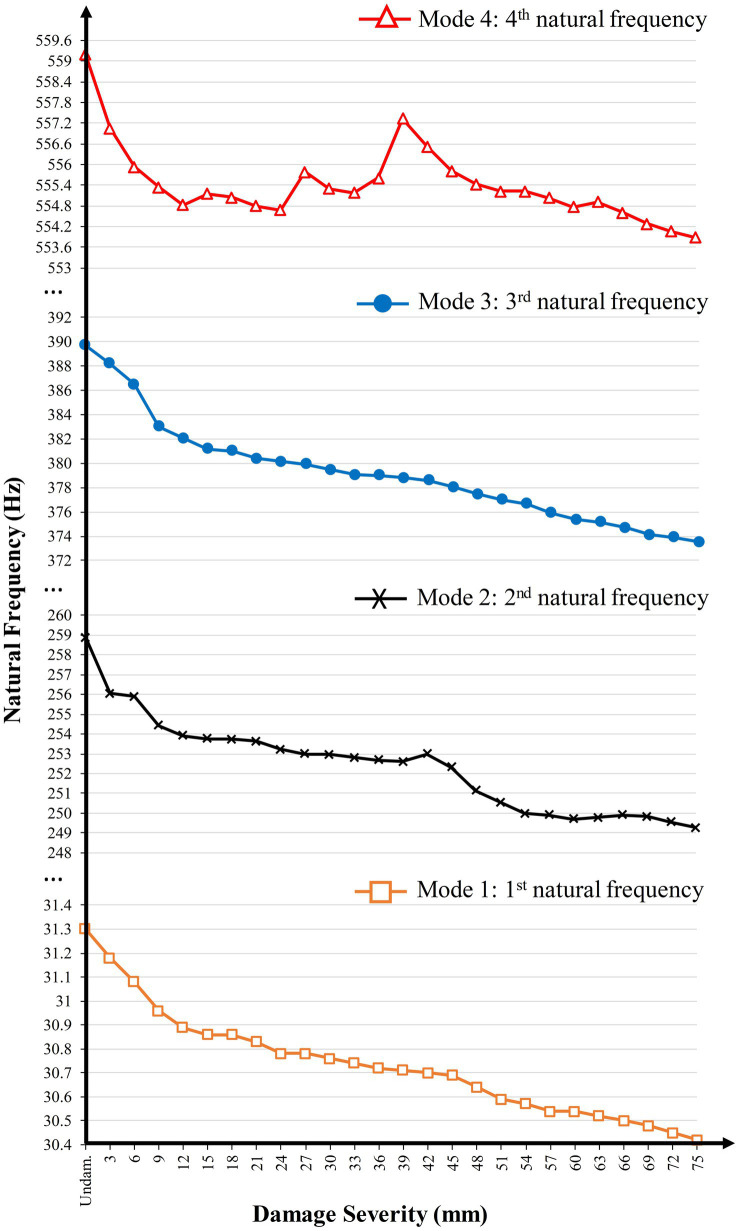
Experimental natural frequency reduction in all damage states of the bridge in 4 modes.

Modal testing was conducted using an intact structure as the reference model to extract the flexural modes. Then, experimental modal analysis continued by introducing several damage scenarios imposed to the test specimen to generate FRF data. Version 10.0 of NVGate software was used in this study as a recorder. Exported FRFs in the frequency domain obtained from experimental modal analysis were used as inputs in the modal analyzer software, ICATS. It should be noted that only flexural modes were considered in this study. The coherence values from NVGate indicated the reliable quality of the measured FRFs. In the next step, ICATS was utilized to extract the structural dynamic parameters from measured vibration data and compute the FRFs by means of curve-fitting extraction process. Figure 2 shows the generated dataset. In this figure, the horizontal axis represents the 26 damage states comprising the healthy case as well as 25 damage scenarios, and the vertical axis denotes the natural frequency values. Accordingly, natural frequencies entirely decreased with increasing damage severity. However, there are also minor fluctuations and low reductions of natural frequencies due to node point locations.

As mentioned before, the ANN, ANN-GA, and ANN-ICA algorithms were applied in this study. The first four modal parameters of undamaged structure along with damaged states and the damage depths acquired from experimental work have been used as the input and target of the aforesaid artificial intelligence networks, respectively. The collected dataset was divided randomly into two subsets, i.e., 80% training and 20% testing sets. Each neural network architecture included different characteristics for training, i.e., types of data, the topology of the network, number of neurons in each layer, weights, activation forms. Therefore, the mentioned principles were important to generate the best network. In this regard, the backpropagation algorithm in a feed-forward network using different topologies was evaluated in this study to achieve the best potential prediction performance. The training process eventually kept on modifying the linking weights until getting an acceptable point. However, the shortcomings of over-fitting as well as inefficient optimum topologies increased the error in the outputs. Consequently, GA and ICA were used in the learning process of ANN to optimize the weights as well as minimize the cost function. To this end, GA was obtained employing the following parameters: the population size and maximum generations set to 150 and 50, crossover and mutation set to 0.5 and 0.35, respectively. Likewise, ICA was obtained by means of the subsequent factors: the number of initial counties set to 100; imperialists set to 15; and coefficient β set to 2.

**Figure 3:**
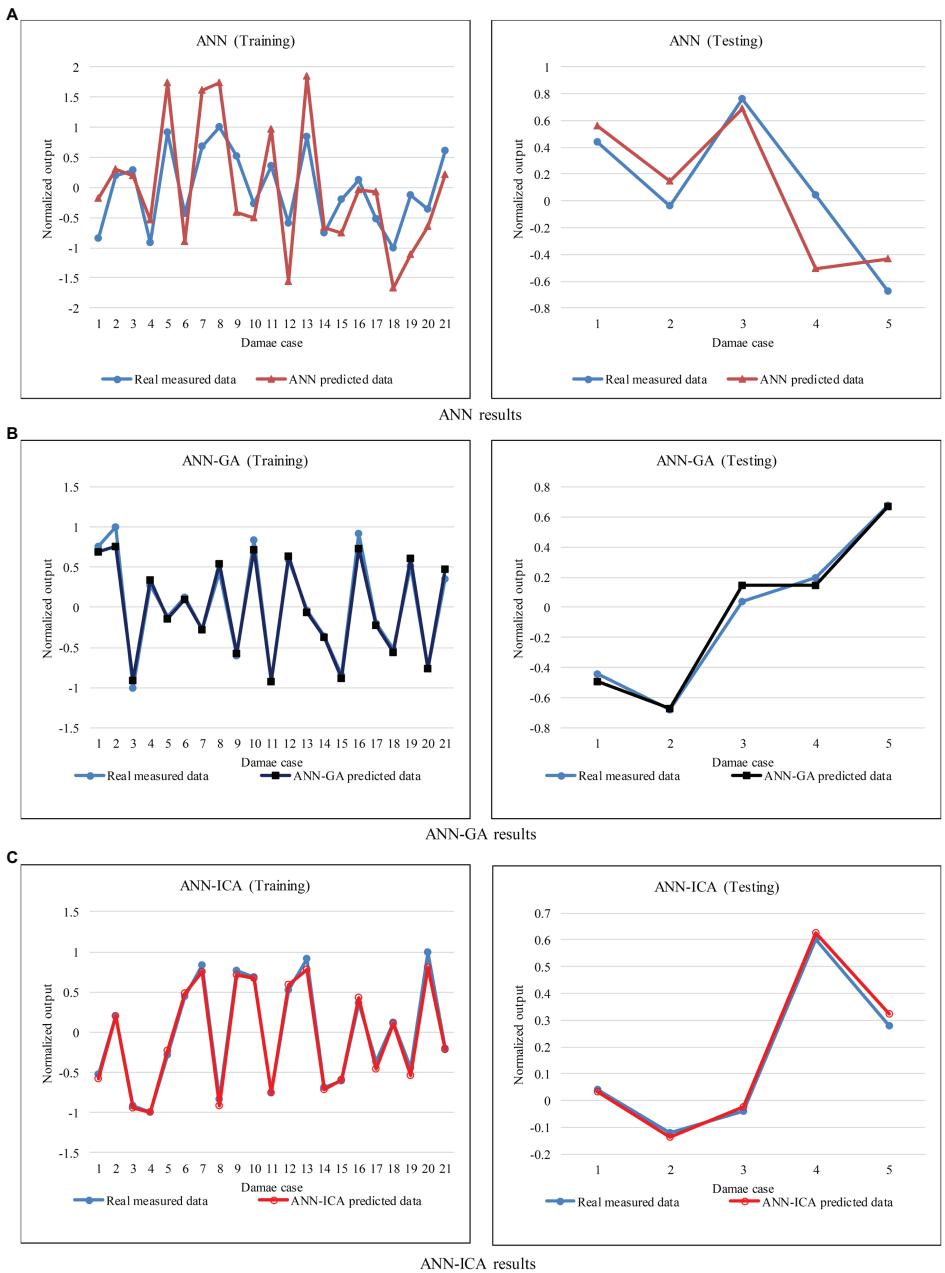
Comparison of results of networks in their training and testing segments, **(A)** ANN, **(B)** ANN-GA, and **(C)** ANN-ICA results.

**Figure 4:**
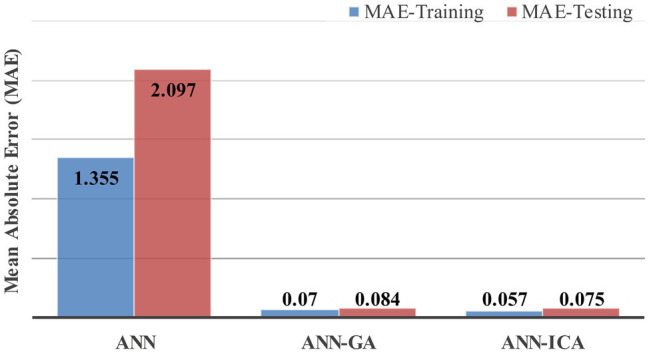
Comparison between the performance of networks.

Figures 3A–C shows a comparison between predicted and measured variables using ANN, ANN-GA, and ANN-ICA at training and testing segments. As it can be seen from the figure, the normalized predicted damage severities were decently fitted to the actual measurements. However, the hybrid networks clearly depict a better fitness in comparison with the pre-developed network. The variance of the estimated result and measured data was considered as network error. In this direction, the Mean Absolute Error (MAE) was evaluated the performance of the patterns, as shown in Figure 4. According to this figure, the most appropriate robustness was succeeded by hybrid algorithms due to enhancing the learning procedure of the ANN utilizing metaheuristic algorithms. Therefore, the performance of algorithms from best to worst achieved by ANN-ICA, ANN-GA, and the pre-developed ANN, respectively. In other words, ICA showed better performance compared to GA.

## Future Work Direction

**Figure 5:**
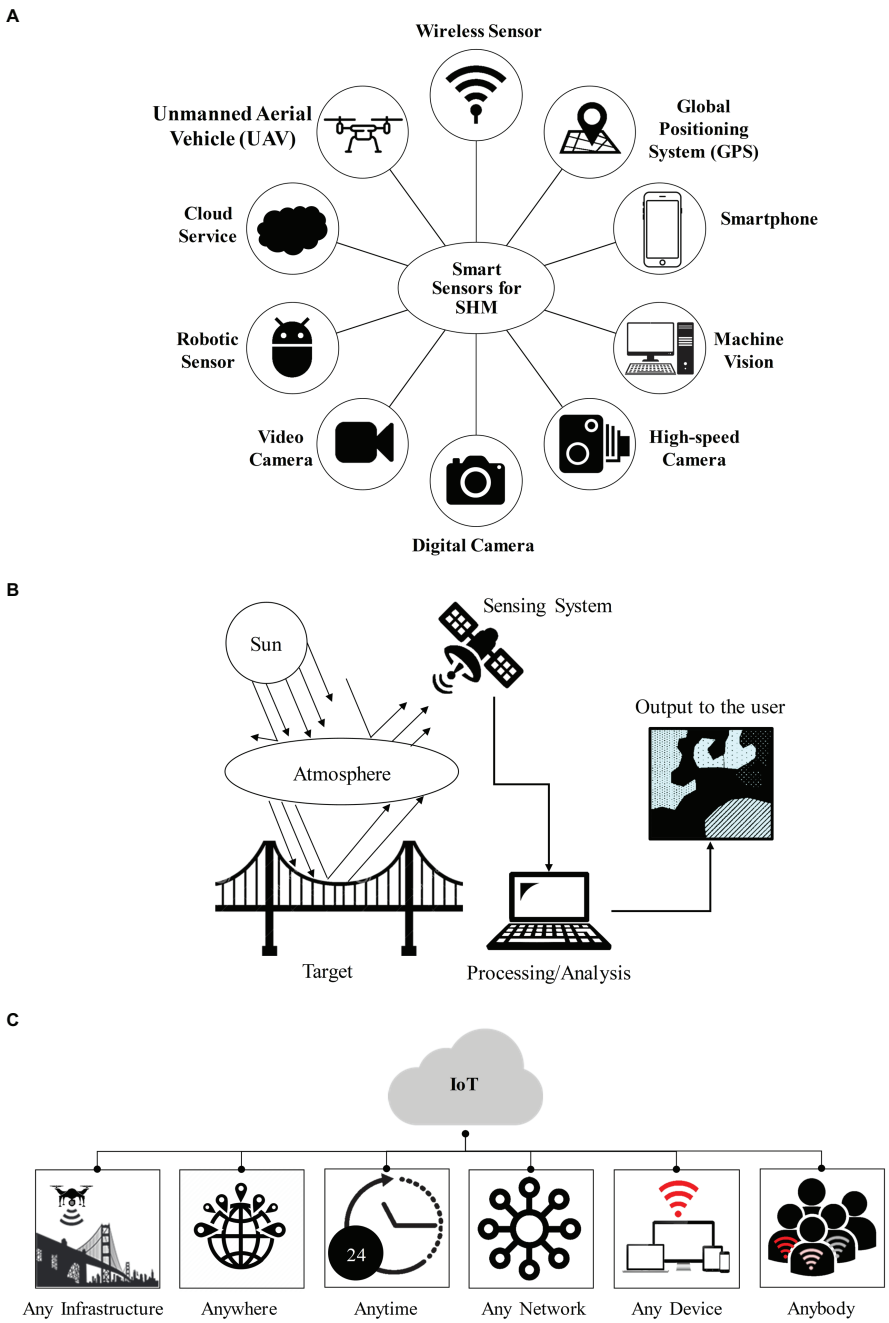
**(A)** Smart sensing technologies for SHM, **(B)** components of a remote sensing system, and **(C)** the concept of IoT.

Remote sensing is defined as the analysis of object properties, area, or phenomenon on the earth’s surface through data acquired from a device that is not in contact with the object, area, or phenomenon under investigation, i.e., terrestrial aircraft and satellites in order to obtain information about the asset (Gordan et al., 2021b). Computer vision-based methods and remote sensing technologies have a direct impact on gaining SHM systems due to their powerful flexibilities, such as wide field of view, non-contact, low cost, and fast response capacities. It is because remote sensing is often applied to monitor near-real-time damage for large-scale events (Ghaedi et al., 2021). With the advancement of modern wireless communication technologies, Internet of Things (IoT) has also become a widely used technology in the field of various intelligent services and applications (Talebkhah et al., 2020, 2021). For example, Wireless Sensor Networks, as the basic layer of IoT, can support real-time and continuous remote sensing data transmission which is based on frequency division multiplexing technology. Therefore, the aforesaid cutting-edge technologies should be combined with SHM systems to upgrade traditional damage detection approaches. Figure 5 presents the schematic of various next-generation sensors for the SHM as well as the components of a remote sensing system and the concept of IoT, respectively.

## Conclusion

More than a billion structures exist on our planet comprising million bridges. A number of these infrastructures are near to or have already exceeded their design life and maintaining their health condition is an engineering optimization problem. Besides, these assets are damage-prone during their service life. This is due to the fact that different external loads can disturb the serviceability and integrity of infrastructures. To overcome such bottlenecks, SHM systems have been used to guarantee the safe functioning of structures in order to make satisfactory decisions on structural maintenance, repair, and rehabilitation. However, conventional SHM approaches cannot be used in crucial decision making for maintenance planning due to overestimating the accuracy of human judgments. On the other hand, the monitoring process in SHM creates lots of data. Classical statistical methods are one of the common tools for knowledge discovery. Nevertheless, they have many drawbacks such as difficulty in assumptions to meet the real work, time consuming, and focusing solely on the simplified quantitative analysis which cannot solve the real world problems. Thus, traditional statistical methods work relatively inefficiently. In order to overcome these problems, sophisticated tools and advanced computing technologies such as artificial intelligence, IoT, and remote sensing can be helpful to handle the qualitative analysis of the complex real world behavior. Therefore, SHM and advanced computational techniques as powerful tools can be significantly used to mitigate the aforesaid concerns by planning scheduled maintenance, control, and management of infrastructures. In this direction, an inverse analysis was conducted using a lab-scale composite bridge deck structure in order to identify the damage severity. To aid the aim, the pre-developed ANN and hybrid ANN integrated with GA and ICA were developed using vibration characteristics obtained from the experimental modal analysis. Then, the performance of models was evaluated by comparing their MAE. Based on the comparison of three employed networks, the aforesaid evolutionary algorithms could improve the prediction error of the pre-developed network by enhancing the learning procedure of the ANN.

## Data Availability Statement

The raw data supporting the conclusions of this article will be made available by the authors, without undue reservation, upon request to the corresponding author.

## Author Contributions

MG: conceptualization, methodology, fabrication of the experimental model, data acquisition, formal analysis, writing-original draft, and validation. OC: writing-review and editing, funding acquisition, and resources. S-RS-Y: investigation and support. ZI: project administration, resources, and funding acquisition. LW: investigation and support. KG: support. All authors contributed to manuscript revision, read, and approved the submitted version.

## Funding

This research was funded by the Universiti Malaya, grant numbers IIRG007A-2019 and IIRG007B-2019.

## Conflict of Interest

The authors declare that the research was conducted in the absence of any commercial or financial relationships that could be construed as a potential conflict of interest.

## Publisher’s Note

All claims expressed in this article are solely those of the authors and do not necessarily represent those of their affiliated organizations, or those of the publisher, the editors and the reviewers. Any product that may be evaluated in this article, or claim that may be made by its manufacturer, is not guaranteed or endorsed by the publisher.
